# COVID-19-related conspiracy beliefs and their determinants among 18 to 45 years old: A cross-sectional study

**DOI:** 10.1097/MD.0000000000030836

**Published:** 2022-09-23

**Authors:** Mohammad A. Al-Qudah, Ala’a F. Al-Shaikh, Shadi Hamouri, Husam Haddad, Samah AbuRashed, Zaid A. Zureikat

**Affiliations:** a Faculty of Medicine, Department of Pathology and Microbiology, Jordan University of Science and Technology, Irbid, Jordan; b Independent Researcher, Amman, Jordan; c Faculty of Medicine, Department of Surgery and Urology, Jordan University of Science and Technology, Irbid, Jordan; d Department of Pathology and Microbiology, Ministry of Health, Amman, Jordan; e Internship Doctor, King Abdullah University Hospital, Jordan University of Science and Technology, Irbid, Jordan; f Department of Radiology, Royal Medical Services, Amman, Jordan.

**Keywords:** conspiracy theories, COVID-19, education, perceived stress

## Abstract

The existence of conspiracy beliefs has been previously linked to multiple individual traits and factors, such as anxiety, lack of information, education, and social factors. This study aims to explore the factors and variables influencing the individual’s susceptibility to conspiratorial thinking, as well as the impact of COVID-19 conspiracy belief on the adoption of public health and social measures. This study explores the factors influencing the susceptibility to conspiratorial thinking and the impact of conspiracy theories on the adoption of public health and social measures. A sample of university students, fresh-graduates, and mid-career professionals between the age of 18 to 45 years old completed an online survey measuring COVID-19 conspiracy beliefs and stress levels. A total of 2417 completed a survey targeting COVID-19 conspiracy beliefs, perceived stress, and demographic information. The results show that COVID-19 conspiracy beliefs were related to education, unemployment, and COVID-19 level of exposure. Meanwhile, conspiracy beliefs had no relation to the individual’s perceived self-reported stress. Higher conspiracy scores were related to lower adoption of preventive measures and increased hesitancy towards COVID-19 vaccination. Lack of knowledge and misinformation actions play a vital role in the generation of conspiracy theories surrounding the COVID-19 pandemic.

## 1. Introduction

Conspiracy beliefs are events narratives that claim the existence of a secret plan to permit ominous endeavors.^[1]^ Meanwhile, medical conspiracy theories are defined as attempts to explain a particular event by a defined group with fixed intentions. Although, the conspiracy theories are mostly inaccurate, but they are not necessarily false.^[2]^

Conspiracy beliefs have been previously associated with four main principles: consequential, emotional, universal, and social.^[3]^ Consequential due to their impact and consequences on health, relationships, and behavior life decisions. An example of this is caregivers’ refusal to vaccinate their children due to misbeliefs and conspiracies surrounding vaccination.^[4,5]^ Conspiracy beliefs are further emotional as the reliance on analytical thinking and arguments tends to decrease the adoption of conspiracy theories.^[1]^ Thus, educational attainment is among the major factors related to the adoption of conspiratorial belief.^[6]^

The third basic principle associated with conspiratorial thinking is universal, which implies that it exists in all communities and is not restricted to certain cultures or time.^[7]^ Despite that, some communities are disproportionally affected by the conspiracy belief. This could be partially related to the fourth principle, which is social. The social phenomenon revolves around the fact that conspiracy theories are usually linked to intergroup conflicts, and their motive to harm a society for another to benefit.^[8]^ Moreover, some scholars relate the increase in the adoption of conspiracy to vulnerability with the feeling of powerlessness as a predictor of the level of conspiratorial belief.^[9]^

Conspiracy theory has been previously linked to multiple individual traits and factors. Among those are the broader conspiratorial belief, dissatisfaction, isolation, and psychological factors, such as anxiety.^[10]^ On the broader level, conspiratorial belief is widespread in impactful crises.^[11]^ It is further increased when there is a lack of information leading to increased curiosity.^[12]^ Thus, a considerable range of conspiracy theories rose during the pandemic. The amount of misinformation, disinformation, and rumors led to the World Health Organization declaring the pandemic as an infodemic.^[13]^ This could be of significant importance as previous reports have shown that conspiracy beliefs and theories could negatively impact the adoption of COVID-19’s preventive and social measures and increase vaccination hesitancy hindering the efforts to contain the pandemic and attenuate its effect.^[14]^

Conspiratorial belief amid COVID-19 is a relatively new topic that has not been holistically addressed, particularly in low- and middle-income countries. This study is an extension of previous studies on conspiracy theories that target to explore the factors and variables influencing the individual’s susceptibility to conspiratorial thinking, as well as the impact of COVID-19 conspiracy belief on the adoption of public health and social measures.

## 2. Methods

### 2.1. Study design

This cross-sectional study used an online survey. The recruitment of participants was through referral sampling, through sharing it with contacts and social media platforms. Targeted population was university students, fresh-graduates, and mid-career professionals between the age of 18 to 45 years old. All cases below the age of 18 years old or above the age of 45 were excluded. The collection tool was a 12-item questionnaire assessing demographics and previous exposure to COVID-19, beliefs surrounding COVID-19, and perceived stress scale. Ethical approval was granted by the institutional review board at Jordan University of Science and Technology.

### 2.2. Measures

#### 2.2.1. Perceived stress scale.

The Perceived Stress Scale (PSS) was used to capture participant’s self-assessment of current stress. The short 4-item version was adopted and used in this study, with answers rating from 0 (never) to 4 (very often). It has been proven that its use in studies relating to conspiratorial beliefs have good predictive validity. The Cronbach’s alpha for the current study was 0.66 which is acceptable in short-item questionnaires. This is close to the reported internal reliability of 0.6 for the four-item abridged scale.

#### 2.2.2. COVID-19 conspiracy beliefs.

Due to the changing nature of circulating conspiracies during the pandemic and its variation between countries, a 9-item COVID-19 conspiracy scale was developed for this study. The scale was based on the most common beliefs circulating in Jordan at the time of data collection and built on previous studies examining similar beliefs.^[15,16]^ Each participant reported the extent to which they endorse each statement on a scale from 1 (strongly agree) to 5 (strongly disagree). The statements included that the pandemic is exaggerated, the virus is bio-engineered, the role of mRNA vaccines in altering people’s DNA, the availability of the vaccine since early pandemic, COVID-19 aims to plant a microchip within the human body, the pandemic is a political manipulation, it aims to increase social isolation, and SARS-CoV-2 aim to increase pharmaceutical profit. Our COVID-19 conspiracy belief scale had a very good Cronbach’s alpha of 0.87.

### 2.3. Analytical approach

The relationship between COVID-19 related conspiracy beliefs and PSS was tested using Pearson correlation. Meanwhile, analysis of variance test was used to compare the level of conspiracy belief across different specialties, education, work level, and sex. All analyses were carried using SPSS (Version 26, IBM SPSS Inc., Chicago, IL).

## 3. Results

### 3.1. Demographic data

A total of 2507 participated in the study during March 2021. Out of which, 95 responses were excluded based on the exclusion criteria giving a total of 2417 participants. As seen in Table 1, the participants were 783 (32.5%) males and 1629 (67.5%) females. The majority of participants were between 18 and 24 years of age; 1254 corresponding to 52.0%. 1409 participants were from different healthcare specialties compared to 1003 non-healthcare specialties’ participants. Around half the sample reported being at their homes during the pandemic, while 28.3% worked or studied from their institutes and 20.6% unemployed.

**Table 1:** Demographic characteristics of the sample (n = 2412).

Characteristics	Number	%
Sex		
Male	783	32.5
Female	1629	67.5
Age		
18–24	1254	52.0
25–34	847	35.1
35–45	311	12.9
Status		
University student (1–3rd year)	692	28.7
University student (4–6th year)	390	16.2
Fresh graduate	406	16.8
Mid-career	924	38.3
Specialty		
Applied health sciences	225	9.3
Dentistry	259	10.7
Medicine and surgery	428	17.7
Nursing	55	2.3
Pharmacy	325	13.5
Other healthcare specialties	117	4.8
Non-healthcare specialties	1003	41.6
Work/study arrangement		
Remotely	1231	51.0
Workplace	683	28.3
Not working/studying	498	20.6

### 3.2. COVID-19 conspiratorial belief

As stated before, the conspiracy belief scale included nine different statements surrounding the COVID-19 pandemic and vaccines. The statement relating the pandemic with microchips installation was the least believed among both healthcare, and non-healthcare specialties, 5.6% and 7.1% respectively. Meanwhile, statements about the virus being exaggerated and bio-engineered yielded the most agreement; 48.8% and 33.6% respectively.

**Figure 1.**
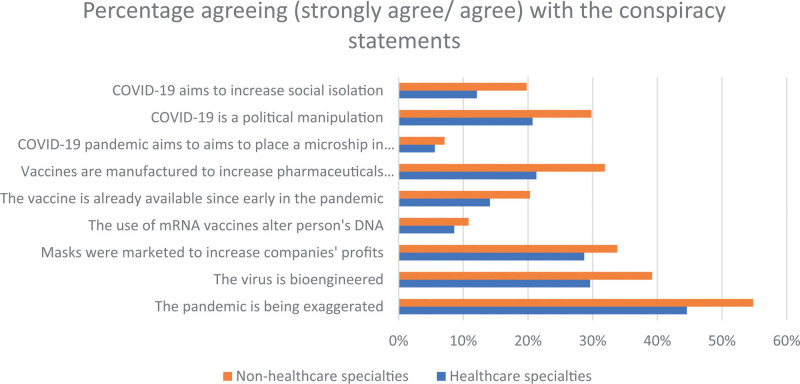
The endorsement of each of the nine conspiracy belief statements for both healthcare and non-healthcare professionals.

Comparing healthcare to non-healthcare specialties, the endorsement of all conspiracy theory statements was lower in healthcare specialties, as seen in Figure 1. The most distinct differences were seen in the statements emphasizing that the pandemic was exaggerated, and vaccines aim to increase pharmaceutical profits; 10.3% and 10.6% respectively. Comparing healthcare specialties, medicine and surgery students and graduates were the least likely to agree with all statements compared to other healthcare specialties.

The reported endorsement of conspiracy beliefs had a mean of (23.25 ± 7.19 SD), on a score range between 9 and 45. The participants’ mean score fell below the midpoint, indicating that the sample does not strongly support conspiracy beliefs. The conspiracy score was significantly contrasting between different career statuses (*F*(3,24) = 5.2, *P* value < .01). post hoc comparisons showed that 4th to 6th year university students had lower conspiracy belief scores compared to 1st to 3rd year university students, fresh graduates, and mid-career professionals (Table 2).

**Table 2:** Post hoc comparison comparing the variation between different career statuses.

(I) Currently you are working/studying	(J) Currently you are working/studying	Mean difference (I–J)	Std. Error	Sig.
University student (1–3rd year)	University student (4–6 year)	1.294*	0.454	0.023
	Fresh graduates (≤2 yr of experience)	−0.102	0.448	0.996
	Mid-career (2–10 yr of experience)	−0.397	0.361	0.688
University student (4–6th year)	University student (1–3rd year)	−1.294*	0.454	0.023
	Fresh graduates (≤2 yr of experience)	−1.397*	0.508	0.031
	Mid-career (2–10 yr of experience)	−1.692*	0.433	0.001
Fresh graduates (≤2 yr of experience)	University student (1–3rd year)	0.102	0.448	0.996
	University student (4–6th year)	1.397*	0.508	0.031
	Mid-career (2–10 yr of experience)	−0.295	0.427	0.900
Mid-career (2–10 yr of experience)	University student (1–3rd year)	0.397	0.361	0.688
	University student (4–6th year)	1.692*	0.433	0.001
	Fresh graduates (≤2 yr of experience)	0.295	0.427	0.900

* Statistically significant mean difference.

Comparing specialties, participants from healthcare specialties had lower COVID-19 conspiracy beliefs compared to non-healthcare specialties (22.37 vs 24.50, *P* value < .001). Among healthcare specialties, medicine and surgery students and graduates had lower conspiracy belief scores compared to all other healthcare specialties (Table 3). All were significant, except for the variation from nursing students, which could be related to the limited number of nursing participants (*P* value < .05).

**Table 3:** COVID-19 conspiracy belief score disaggregated by specialty.

Multiple comparisons				
Dependent variable:				
Tukey HSD				
(I) Specialty		Mean difference (I–J)	Std. Error	Sig.
Applied health sciences	Dentistry	1.259	0.643	0.443
	Medicine and surgery	3.106*	0.581	0.000
	Nursing	1.201	1.062	0.919
	Pharmacy	0.921	0.612	0.742
Dentistry	Applied health sciences	−1.259	0.643	0.443
	Medicine and surgery	1.847*	0.556	0.016
	Nursing	−0.058	1.048	1.000
	Pharmacy	−0.337	0.588	0.998
Medicine and surgery	Applied health sciences	−3.106*	0.581	0.000
	Dentistry	−1.847*	0.556	0.016
	Nursing	−1.905	1.011	0.491
	Pharmacy	−2.184*	0.519	0.001
Nursing	Applied health sciences	−1.201	1.062	0.919
	Dentistry	0.058	1.048	1.000
	Medicine and surgery	1.905	1.011	0.491
	Pharmacy	−0.279	1.029	1.000
Pharmacy	Applied health sciences	−0.921	0.612	0.742
	Dentistry	0.337	0.588	0.998
	Medicine and surgery	2.184*	0.519	0.001
	Nursing	0.279	1.029	1.000

* The mean difference is significant at the 0.05 level.

There was no significant variation in COVID-19 conspiratorial beliefs scores based on sex or age. Meanwhile, unemployment was significantly associated with higher COVID-19 conspiratorial beliefs. Similarly, exposure played a role in endorsing conspiracy theories surrounding COVID-19 with significant variation seen between people who were previously infected or experienced the injury of a beloved one, to those who did not know any previously infected individuals (*P* value < .01).

### 3.3. Perceived stress scale

The PSS scores had a mean of (8.0 ± 2.9 SD), on a range of 0 to 16. The mean fell in the middle range of the score, indicating that the sample had modest levels of stress. Healthcare specialties students and graduates had significantly higher levels of stress compared to non-healthcare specialties (mean 8.27 vs 7.67 respectively, *P* value < .01).

Meanwhile, females had significantly higher scores than male participants, 8.3 vs 7.2 respectively (*P* value < .001). Age showed an effect on the PSS score, with a decrease in mean score in older age groups, *P* value < .01. Mid-career professionals also had significant variation in their PSS scores compared to all other entities reporting lower PSS scores (*P* value < .001). Similarly, participants working or studying from their institutes reported significantly lower PSS scores (*P* value < .001). The PSS scores were unrelated to COVID-19 conspiracy scores or exposure.

### 3.4. Scores and preventive measures

While PSS scores did not impact people’s beliefs and decisions regarding mask-wearing, physical distancing, or COVID-19 vaccination, COVID-19 conspiracy scores were significantly higher among people who believe that wearing masks and physical distancing doesn’t impact virus spread (*P* value < .05). Moreover, people with higher conspiracy belief scores were less willing to take COVID-19 vaccines (*P* value < .05).

## 4. Discussion

The results in this study supported previous literature suggesting that educational attainment could significantly impact the adoption of conspiratorial beliefs.^[17]^ This is seen through the mean scores indicating feeble support of COVID-19 conspiracy theories among the current sample of well-educated bachelor-level professionals. This agrees with previous studies reporting a higher level of conspiratorial beliefs among the less-educated population. This is commonly related to the absence of analytical thinking and improved cognitive skills.^[17,18]^

Healthcare students and professionals were reported to have significantly higher stress scores than non-healthcare workers, agreeing with previous studies reporting similar results among healthcare which were related to increased risk and stressful working conditions, particularly amid the COVID-19 pandemic.^[19]^ Despite reported higher stress levels, healthcare professionals were less endorsing of conspiracy theories. It could be partially related to the higher level of exposure, medical knowledge, access to more reliable resources, and improved analytical thinking which combat one of the reported motives of conspiratorial beliefs; deficiency in available information.^[12,15]^ Among healthcare workers, medicine and surgery professionals were significantly less endorsing of COVID-19 conspiracy theories. This supports the role of lack of information in adopting conspiratorial belief to fulfill people’s curiosity.^[12]^

Unemployment was another triggering factor for conspiracy theories surrounding the COVID-19 pandemic. Lack of empowerment, confidence in authorities, and mistrust are among other contributing factors that could influence disadvantaged persons to adopt conspiratorial thinking.^[20,21]^ It was also previously related to the impact of employment security in the formation of conspiracy theories.^[22]^ However, this disagrees with previous results indicating no role for unemployment in shaping such beliefs.^[23]^

Based on previous literature, stress was anticipated to be associated with the endorsement of conspiracy theories.^[24]^ Nonetheless, this was not observed in the current study contrary to a recent study among university students reporting a significant association between anxiety levels and conspiracy belief surrounding COVID-19.^[25]^ However, the current results are similar to a recent study reporting a similar lack of association in a sample of 18 to 25 year old participants. While some scholars might relate this to the lower perceived threat due to the relatively younger sample, others may argue that it is related to protection motivation theory since the data collection was during an upsurge in the number of cases.^[16,26]^ Previous exposure to COVID-19, whether being infected or the illness of a beloved one, was another determinant of COVID-19 conspiracy theories’ endorsement. This agrees with previous studies reporting lower conspiracy scores in countries that were highly affected by the pandemic.^[16]^

Population with higher COVID-19 conspiracy beliefs were less likely and willing to practice the widely communicated public health and social measures of mask-wearing, physical distancing in addition to lower acceptance of COVID-19 vaccination. Thus, conspiracy belief scores can predict the degree of resistance to preventive measures and vaccination. This agrees with previous studies reporting similar findings in the United States.^[14]^

## 5. Limitations

This study suffers from several limitations. Using online surveys could lead to sampling and acquiescence bias. In a trial to minimize sampling bias, the questionnaire was distributed through multiple platforms and channels increasing its visibility. Another limitation is the higher rate of female respondents and the limited number of nursing professionals in the study.

## 6. Conclusion

The adoption of conspiracy beliefs surrounding COVID-19 could be related to the level of education, unemployment, and exposure to COVID-19. Correlations further showed that it is linked with the lack of information and thus thrives in the presence of scientific ambiguity and in time of pandemics. The flawed beliefs might be associated with lower tendency to adopt public health measures. Thus, awareness and communication campaign should consider addressing the most widely communicated conspiracy theories in their plans.

## Author contributions

**Conceptualization:** Mohammad A. Al-Qudah, Shadi Hamouri.

**Data curation:** Samah AbuRashed.

**Formal analysis:** Ala’a F. Al-Shaikh.

**Investigation:** Samah AbuRashed.

**Methodology:** Ala’a F. Al-Shaikh, Husam Haddad.

**Project administration:** Husam Haddad, Samah AbuRashed.

**Supervision:** Mohammad A. Al-Qudah.

**Writing – original draft:** Ala’a F. Al-Shaikh, Shadi Hamouri, Husam Haddad.

**Writing – review & editing:** Mohammad A. Al-Qudah, Ala’a F. Al-Shaikh, Zaid A. Zureikat.
